# Automatic Visual Tracking and Social Behaviour Analysis with Multiple Mice

**DOI:** 10.1371/journal.pone.0074557

**Published:** 2013-09-16

**Authors:** Luca Giancardo, Diego Sona, Huiping Huang, Sara Sannino, Francesca Managò, Diego Scheggia, Francesco Papaleo, Vittorio Murino

**Affiliations:** 1 Pattern Analysis and Computer Vision, Istituto Italiano di Tecnologia, Genova, Italy; 2 Neuroscience and Brain Technologies, Istituto Italiano di Tecnologia, Genova, Italy; 3 Dipartimento di Scienze del Farmaco, Università degli Studi di Padova, Padova, Italy; University of Queensland, Australia

## Abstract

Social interactions are made of complex behavioural actions that might be found in all mammalians, including humans and rodents. Recently, mouse models are increasingly being used in preclinical research to understand the biological basis of social-related pathologies or abnormalities. However, reliable and flexible automatic systems able to precisely quantify social behavioural interactions of multiple mice are still missing. Here, we present a system built on two components. A module able to accurately track the position of multiple interacting mice from videos, regardless of their fur colour or light settings, and a module that automatically characterise social and non-social behaviours. The behavioural analysis is obtained by deriving a new set of specialised spatio-temporal features from the tracker output. These features are further employed by a learning-by-example classifier, which predicts for each frame and for each mouse in the cage one of the behaviours learnt from the examples given by the experimenters. The system is validated on an extensive set of experimental trials involving multiple mice in an open arena. In a first evaluation we compare the classifier output with the independent evaluation of two human graders, obtaining comparable results. Then, we show the applicability of our technique to multiple mice settings, using up to four interacting mice. The system is also compared with a solution recently proposed in the literature that, similarly to us, addresses the problem with a learning-by-examples approach. Finally, we further validated our automatic system to differentiate between C57B/6J (a commonly used reference inbred strain) and BTBR T+tf/J (a mouse model for autism spectrum disorders). Overall, these data demonstrate the validity and effectiveness of this new machine learning system in the detection of social and non-social behaviours in multiple (>2) interacting mice, and its versatility to deal with different experimental settings and scenarios.

## Introduction

Social abnormalities in mental illnesses profoundly affect the life quality of patients and their families, and still limited therapeutical strategies are available for these behavioural diseases [1], [2]. Mental disorders characterised by severe social anomalies such as schizophrenia and autism have a strong genetic heritability. However, the complexity of human genetics, the clinical heterogeneity, the uncontrollable impact of gene-gene and gene-environment interactions have hindered our understanding of the neurobiological basis of social-related disorders and the development of effective treatments.

Mice are a social species engaging in high degrees of social interactions [3], [4]. Moreover, genetically modified mice are now commonly generated and used, making them a unique tool to elucidate the links between genes and behaviour [5], and thus to understand the neurobiological basis of social abnormalities in psychiatric disorders. A central issue in the analysis of complex social behaviours is the reliable and objective investigation of specific behavioural parameters, which might span for extended periods. In such investigations, a manual scoring of the social interactions is still the preponderant experimental bottleneck [6], [7]. Indeed, manual scoring suffers from a number of limitations such as scarce replicability and lack of standardisation. Moreover, it is extremely challenging and time consuming to visually follow subtle and composite social behaviours, especially when multiple animals are involved. As a consequence, more explanatory long-lasting and/or large-scale studies are still unaffordable. Hence, unless technological innovation is introduced to facilitate the analysis, our ability to link genetics and complex social behaviours in mice will remain limited in the extent of the experimental protocols, which in turn will limit the translational advances in psychiatric medicine.

Hence, there is an increasing interest on the development of systems for automated behaviour analysis from videos. Aiming at the above issues, this work proposes a computational framework integrating a tracking algorithm able to simultaneously track multiple mice and a new automatic method for classifying behaviours of multiple interacting mice using a learning-by-examples approach.

In this type of problems, the first issue to be tackled is the “multiple animal tracking”, i.e., the automatic detection of the positions of multiple mice along time. A frequently adopted tracking solution is based on particle filter modelling extended to multiple targets. An early example of this type is the algorithm devised by Khan et al. [8] which was tested on ant tracking. A similar approach was applied to mice by exploiting their slowly changing contour by imaging the cage through a side view [9], [10]. Pistori et al. [11] adopted a particle filtering approach with a variation on the observation model in order to track multiple mice from top view. However, the system was evaluated on white mice on a black background with a coarse position estimate. An attempt to reliably monitor multiple mice in a single cage was made using radio transmitters inserted under the skin and then recorded by detection coils [12]. However, this system is invasive for the tested subjects, it has low spatial resolution and it exposes the animals to a continuous electro-magnetic field.

The second issue to be addressed is the automatic analysis of mouse behaviour. In the computer vision literature, a large variety of methods for human action/activity recognition are proposed. In this context, various general purpose spatio-temporal descriptors were introduced for activity recognition tasks, such as HOG/HOF [13], HOG3D [14], eSURF [15] and hierarchical spatio-temporal features [16], to quote a few. Some of these descriptors were applied as the basic encoding step for single mouse behaviour analysis in home cages [17], [18] followed by a classification stage or for more complex behaviours [19], [20]. However, they were limited to *single* mouse processing, making them inapplicable for the analysis of multifaceted social disorders [21].

More recently, two new methods have been developed to monitor the behaviour of two mice in the same cage in a resident/intruder task [22], [23]. Specifically, De Chaumont et al. [22] developed a model exploiting physics-based principles on a set of geometrical primitives to track the position of two mice and to monitor their interactions. The interactions were described by a fixed set of rules, based on relative mice positions and accelerations and on some numerical parameters that need to be manually set in the software through a trial-and-error phase. More importantly, their system does not actually classify social behaviour with respect to a set of standard classes as given by a human grader and defined in many social behaviour studies, but rather it detects and quantifies very specific mouse configurations such as, for instance “mice distance is less than a given threshold”, “mouse A gets to mouse B and B escapes with a given distance threshold” or “mouse A is behind B and nearer than a given threshold”. While the above precisely defined actions are very objective (i.e., unrelated to a subjective human interpretation), they might not correspond to a required personalised scoring of social behaviours by a grader with some specific needs. In fact, all behaviours need to be expressed in terms of a predefined set of basic rules. Additionally, all these rules are hard-coded in the system, making the addition to the system of any new behaviour difficult and error prone. Similar approaches appear to be adopted by commercial systems developed by two companies: Clever System, Inc. (http://cleversysinc.com) and Noldus (http://www.noldus.com). However, the inner details of the algorithms employed are not fully disclosed.

Alternatively, Burgos-Artizzu et al. [23] adopted an approach based on machine learning, where the behaviour is learned from examples given by experimenters. Initially, the system tracks mouse positions with an undisclosed algorithm, then the video is analysed extracting a series of general purpose features borrowed from recent computer vision literature. Finally, a machine learning model (AdaBoost with temporal context features [13]) is trained to classify the behaviour of the mouse emulating a human grader. This approach has various advantages. Mouse behaviours can be expressed via visual examples (sequences of video) given by the behavioural scientist rather than by hard-coded numerical parameters. As a result, it is easy to extend the system with new behaviours according to the experimenters needs, moreover, the output has a straightforward interpretation and it can be directly compared to previous manual human-based scorings. Despite this, all these systems are not designed to generalise over multiple mice interactions or different experimental settings. In particular, [23] heavily relies on full frame image-level measures (features), which can only describe the global behaviour occurrences in the arena, hence, only one action can be associated to each frame in the video. This means that only experiments with two mice can be addressed, where one of the two is considered active and the other passive. As a matter of fact, the above image-level features cannot be used to classify all possible interactions of each mouse when more than two mice are involved.

In this paper, we overcome these limitations by proposing an integrated automatic system for the analysis of social interactions, which is characterised by a great adaptability to the expert needs. The idea is to allow for multiple mouse tracking in a freely moving environment, followed by the continuous monitoring of their interactions. Similarly to the system proposed in [23] all classes of interaction are learned from examples given (i.e., annotated) by behavioural scientists. In our system, however, the social interactions are detected on a mouse-by-mouse basis and then appropriately combined in order to obtain all different behaviours allowing either global analyses in the arena or a specific finer analysis of a particular mouse. A further learning-based approach overcoming the limitation on the number of processed mice has been very recently proposed by Kabras et al. [24]. This system focuses on behaviour classification taking advantage of the temporal information, however, it differs from our proposal both for the implemented features and for the classification methods. Actually, instead of encoding the temporal information in the data representation by using a set of temporal features, our system directly processes this information into an ad hoc classification architecture specifically designed to manage temporal relationships. Moreover, our algorithm exploits the extra thermic information acquired by the thermal camera to improve the identity tracking of the mice.

Similarly to the above methods, tracking is the first fundamental step of the process. This task was addressed by developing a robust processing pipeline, composed of blob detection, shape segmentation, refinement and matching modules. This is a challenging task due to the frequent occlusions, the non-rigid shapes to deal with, and the similar appearances of the animals. Animal tracking is then followed by an automatic behavioural classification algorithm based on the *random forests* [25], which is a theoretical framework grounded on a mixture of *decision trees* [26] combining the concept of *boosting* [27] and *random subspaces* [28]. Indeed, while being a general-purpose classifier with excellent performance, random forests have recently gained traction as an effective method for automatic human action recognition, obtaining excellent results in various public datasets. These works can be roughly divided into methods that first track the movement and the appearance of regions of interest and then perform action classification [29]–[31], and integrated frameworks that combine tracking and classification [32], [33]. However, the final purpose of these methods is mainly to detect the occurrence of an action in a video sequence rather than evaluate its global duration. Additionally, none of them considers the detection of social actions of multiple interacting subjects. These two aspects are an absolute requirement for mice social activity classification, in fact, each mouse needs to be constantly monitored in relation to all other mice in a computationally efficient manner and the action duration is one of the main tools to evaluate the results from a neurological perspective.

The original activity classification method presented here extends the random forest approach to the multi-frame case, exploiting the temporal information at the level of the trees rather than at the feature level (i.e., time is encoded with an ensemble of decision tree rather than with features concatenation), hence providing the continuous estimation of the activities of the mice. The multi-frame perspective of the designed classifier allows to exploit the temporal information in order to recognize behaviours that can be understood only considering a certain time span instead of single frames. To summarize, the presented method has several peculiarities as compared to previous systems, which makes it original with respect to the state of the art. First, it is not based on predefined hard-wired (i.e., manually encoded) rules, so it is more directly comparable to manual human-based scorings; second, it can be extended to any kind of behaviour, depending on the need of the experimenters; and third, it can be applied to different experimental settings and scenarios involving 2 or more mice. To prove the validity of our approach, the proposed system was evaluated on about 6.5 hours of social interaction labelled by two independent graders. The agreement between grader and classifier is in line with the agreement among the graders. Additionally, our classification approach was compared with the classification strategy of Burgos-Artizzu et al. [23] obtaining higher performance. The datasets employed in this paper are freely available for research purposes.

## Materials

### Ethics Statement

All procedures were approved by the Italian Ministry of Health (permit n. 230/2009-B) and strictly adhere to the recommendations in the Guide for the Care and Use of Laboratory Animals of the National Institutes of Health.

### Experimental Setup

All experiments were carried out in an empty open arena with opaque grey walls (40×40×40 cm) and dimly illuminated by overhead red lightning (mainly to facilitate mice handling by the experimenter). Mice were monitored by an infrared camera mounted 1.5 m above the arena. The camera was a FLIR A315 capable of a spatial resolution of 320×240 px at 30fps, with a thermal sensitivity of <0.05°C at+30°C (according to the producer) that allows full video recording even in full darkness. This set-up allows for video tracking under any light conditions. Indeed, dark conditions are less stressful for mice and correspond to their more active phase [34]. All the animals were recorded without any external artificial tagging (such as coloured paintings).

### Datasets

The method was evaluated using two datasets specifically collected to test the system. “Dataset A” is composed of a set of 4 videos recorded at different points in time with different animals, for a total of 2h30min worth of video monitoring the social behaviour of three interacting animals (“dataA-1”, “dataA-2” and “dataA-3”) and 1h worth of video monitoring the interaction of two animals (“dataA-4”), with a total of 11 different animals. The data was analysed and manually labelled by multiple experts. This dataset was therefore used as ground truth in various evaluation aspects both for mice tracking and for behaviour classification.

Specifically, the ground truth to test the mice tracker was compiled by three behavioural scientists who corrected the initial mice identity estimates made by the tracking algorithm. Specifically, we developed a tool allowing the manual correction of identities determined by the tracking system. In particular, the mice matching module (see below) is enriched with a sanity-check index that reveals possible mistracking due to mice proximity. Using this index, the tool for the identities correction shows the video at high velocity, slowing down only when the sanity index reveals possible identity switches. Hence, the tool allows stopping the video in any moment in order to correct the mice identity. This tool allowed to generate the ground truth in a very short time.

Two independent graders who previously agreed on the number and types of behaviours then compiled the behaviour ground truth. The identity of each mouse was superimposed on the video, allowing a precise scoring on a frame-by-frame basis with ANVIL (a open source video annotation tool [35]). The scoring was done on multiple mice interacting in the open field arena, however, their social behaviours were evaluated using a pair-by-pair strategy, i.e., the movie was multiply annotated, each time for a different pair of mice. In each pair, there was a reference mouse performing the actions and a passive target mouse. By allowing graders to concentrate on two mice at a time, the precision of the scoring task was preserved and a total of ∼6 h 30 min of social behaviours were labelled by each grader. The behavioural labelling required an average of 6 hours per grader to fully analyse a single pair of mice over 30 minutes video.

**Figure 1:**
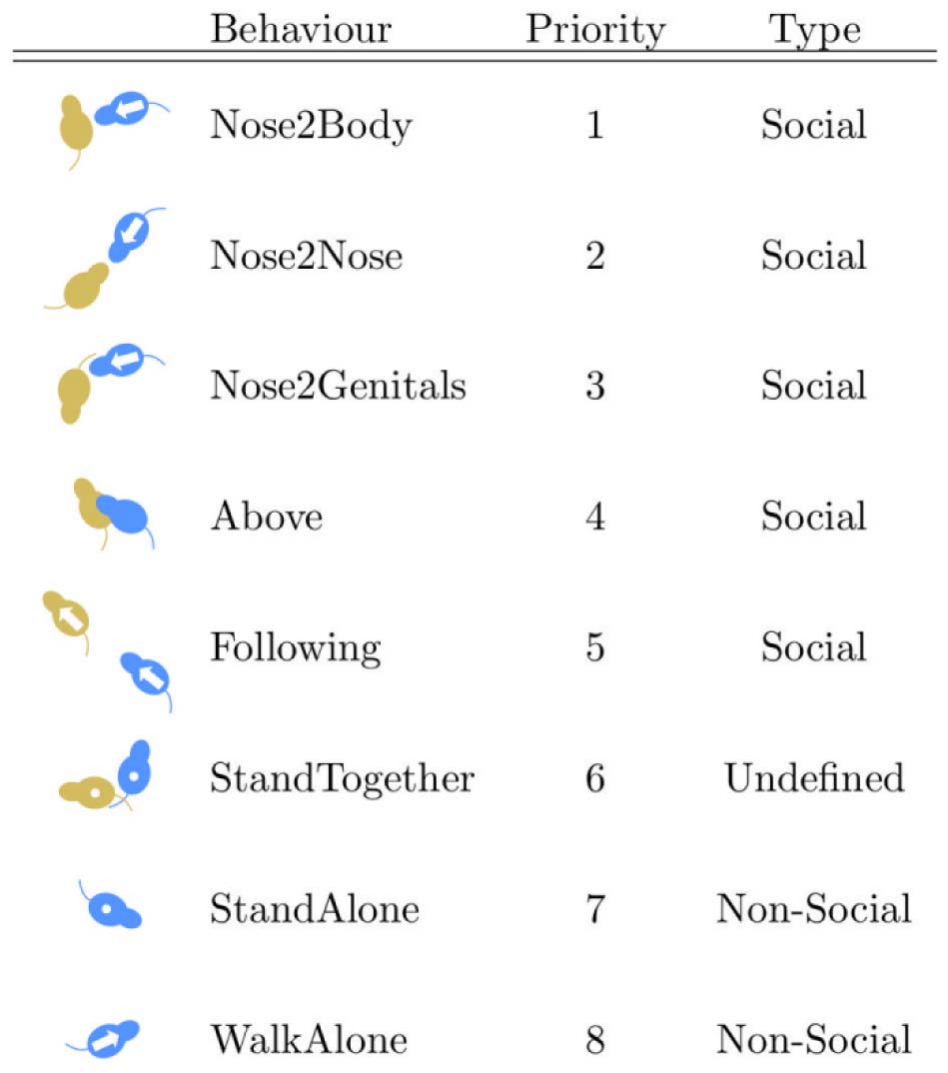
Behaviour priorities employed to combine all pairwise classifiers h*^α^* ^⇒*β*^ in order to describe the behaviour of a mouse with a single class. “1” is the highest priority. The blue mouse is the one actively performing the action. An arrow indicates movement, a circle the lack thereof.

Specifically, eight different mutually exclusive classes of mouse behaviours were considered (see Fig. 1), which were determined as the most reliable parameters used by human graders to score mice social behaviours and that have been previously used in former manual scorings [36]. The definition of the postures were agreed among the graders resulting in a series of guidelines that were followed by the human experimenters to label pieces of video with the behaviours to be then used to train the automatic classifier and test its quality:

**Nose2Body** (Body Sniffing) The nose of the mouse under analysis points towards the body of a conspecific at a distance of less than 0.5 cm or directly touches it.

**Nose2Nose** (Head Sniffing) The nose of the mouse under analysis points towards the nose of a conspecific at a distance of less than 0.5 cm or directly touches it.

**Nose2Genitals** (Anogenital Sniffing) The nose of the mouse under analysis points towards the genitals of a conspecific at a distance of less than 0.5 cm or directly touches it.

**Above** (Crawling) The mouse under analysis crawls over or under a conspecific. **Following** The mouse under analysis walks on the same direction of a conspecific at a distance of less than 10 cm.

**WalkAlone** The mouse under analysis walks in the open field arena without being involved in any of the above-described behaviours.

**StandAlone** The mouse under analysis does not walk and has a distance from any other conspecific of more than 10 cm.

**StandTogether** The mouse under analysis does not walk, it is in contact or at a distance of less than 3 cm from a conspecific, and the two mice have their heads pointing towards any direction other than towards the other mouse.

“Dataset B” is composed of 9 recording sessions of one hour each. Two classes of animals were recorded, the C57BL/6J wild type mouse (7 sessions) and the BTBR T+tf/J mouse (two sessions). BTBR is a mouse strain widely used to model autism-like behaviours, which is known to display a reduced social interaction with its conspecifics [37]. Mice belonging to different strains were not concurrently placed in the same arena for a total of 22 different animals. The experimental setups comprise of 7 sessions with two concurrent mice (C57BL/6J and BTBR) and 2 sessions with four concurrent mice (all C57BL/6J) in the same arena. This dataset was left untouched by graders and it was analysed uniquely by our algorithms without human intervention. This allowed us to have an empirical evaluation of the algorithm performance with a new, larger and diverse set of data. The datasets are freely available to the research community at this URL: http://www.iit.it/en/pavis/mice.html.

## Methodology

**Figure 2:**
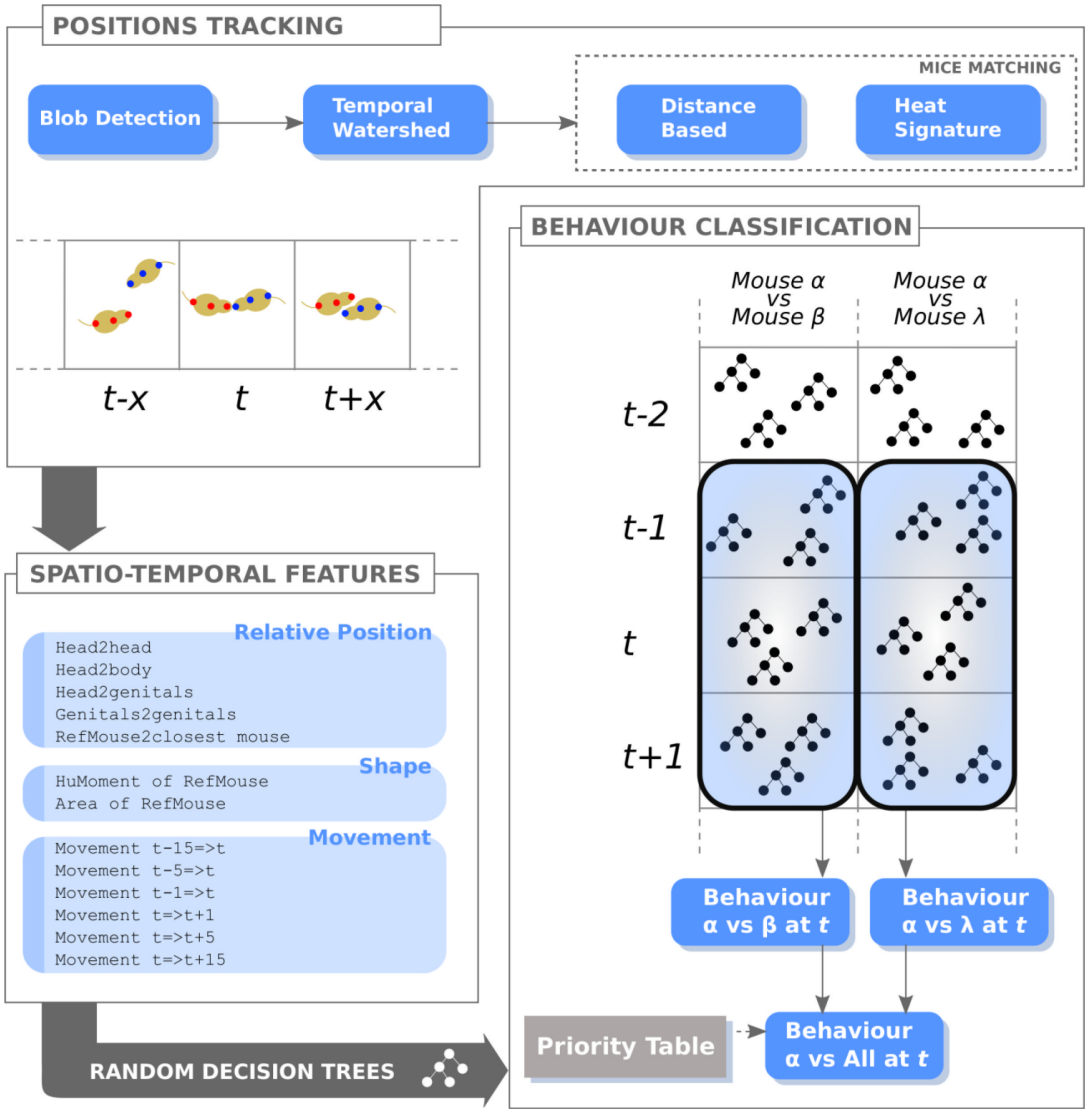
Algorithm diagram summarizing the behaviour classification phases. The “Position Tracking” is composed of a pipeline of three modules. The blob detection module initialises the system, estimates the foreground shapes (i.e. it locates possible mice), and filters out unfeasible structures; the temporal watershed module identifies mouse positions and shapes, and their directionality; the mice matching module tracks the identities of each mouse. Then, a feature vector composed of 13 measurements describes relative position, movement and attitude of mice for all possible pairs. Finally, the continuous action description for the mice is generated thanks to our Temporal Random Forest approach, which evaluates ensembles of decision trees through time.

The proposed method builds upon two main ingredients: mice tracking and behaviour classification. The former module, recently proposed by our group [38], aims at dynamically tracking mouse positions and delineating their shapes through a non-rigid body segmentation in time. The latter exploits the information generated by the tracker to associate the behaviours to each mouse on the basis of rules implicitly provided by the human graders with examples. Figure 2, gives a visual overview of the whole approach.

### Mice Tracking

The tracking algorithm is composed of a pipeline of three modules. The *blob detection* module initialises the system, estimates the foreground shapes (i.e. it locates possible mice), and filters out unfeasible structures; the *temporal watershed* module identifies mouse positions and shapes, and their directionality; finally, the *mice matching* module tracks the identities of each mouse. In this last module, two strategies are employed; one based on the mouse displacement, the other based on a dynamic heat model independent from the position of the mouse. The choice of the strategy is dictated by an automatic sanity check based on a continuous statistical tracking of mouse shape distribution.

### Blob Detection

**Figure 3:**
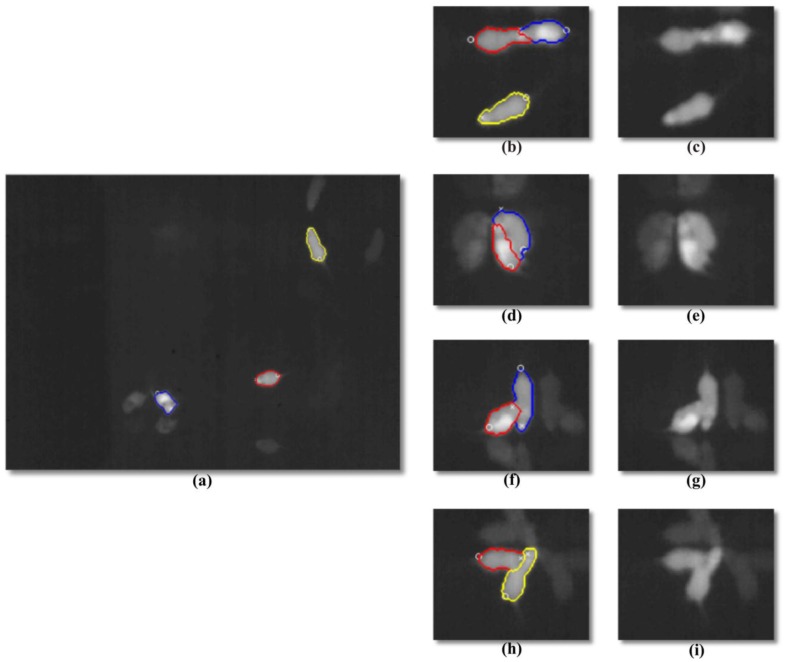
Examples of challenging mice interactions. The red, blue and yellow lines represent the contours detected by the tracking algorithm. (a) The whole mice arena; (b,d,f,h) details of the algorithm output; (c,e,g,i) unprocessed IR details.



The main aim of this module is to separate the mice from the background. As shown in Fig. 3(a), the background includes static elements such as the mouse arena itself, bedding, and some dynamic components such as excretory products and infrared reflections on the cage walls. The foreground separation is done by thresholding all pixels above a limit 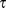 determined from the cumulative distribution function of the temperature histogram 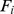 estimated over a few seconds as follows:

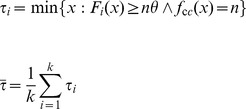
(1)where 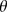 is the expected size of the smallest mouse in pixels, 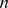 is the number of mice in the arena, 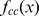 is the number of connected components found (with the method described in [39]) using 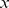 as threshold and 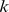 is the number of frames employed for the initialization. The window of 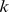 frames used to compute the threshold is slid from the beginning of the video till the condition in Eq. (1) is satisfied for all 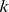 frames in the window. This equation describes a condition in which the system finds for each frame a number of foreground blobs equal to the expected number of mice, and with a total area comparable to the average total mice dimension.

Once the threshold has been determined following Eq. (1), it can be used to find the foreground in all following video frames. The binary image 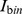 obtained after thresholding is improved with a series of fast morphological operations [40]: the mice tails (frequently disappearing from the video) are removed as 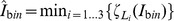 where 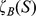 is an opening operation, 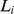 is a 3-dimensional vector containing a 5px linear structuring element rotated at 0°, 45° and 90°.

The connected component analysis described in [39] is performed to estimate location and number of blobs representing the mice bodies. If the number of blobs detected and the number of mice in the arena disagree due to a thresholding failure, the module try to automatically correct the result. If the number of blobs is greater than the number of mice, the image 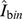 is iteratively dilated until obtaining the expected number of blobs. If this is not the case, the system is re-initialised using the following frames. This might cause some frames to remain unlabelled and hence discarded from the further analysis. In case the number of detected blobs is smaller than the number of mice, the temporal watershed module (described below) is involved in disambiguating the blobs.

### Temporal Watershed

Whenever mice are not in direct contact between each other, their bodies can be easily segmented off the background by the blob detection module. The temperature contrast between mice and background is substantial, therefore, their individual shapes are well-delineated. When they are touching each other, instead, a single blob is detected for multiple bodies. In order to deal with these occlusions, we developed a method called “temporal watershed” which disambiguates the mice, thanks to an extension of the classical watershed segmentation by exploiting the temporal information in an Expectation Maximization (*EM*) framework.

The watershed segmentation [41], as other seed based methods, partitions an image (a video frame in our case) via clues given by two or more labelled areas, i.e. the seeds, whose shapes and positions are essential for a correct segmentation. We employed two types of seeds: one to model the background and the other to model the mice. The background seed is the morphological eroded version of the inverted 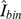; the seed for a mouse 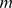 is a linear structuring element made of three points. Specifically, the position of each mouse 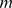 is summarised by a 6-dimensional vector 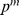, which contains the 2D coordinates of 3 points: nose, genitals and mouse centroid (determined via first order moments). The length (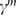) and the position vector (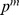) of each mouse 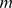 are therefore dynamically calculated using an EM algorithm. More specifically, after the *initialization*, an *expectation* (*E*) and a *maximization* (*M*) step are iterated, estimating the temporal watershed parameters (the mice seeds and lengths) until convergence:

**Initialization:** the seed and the corresponding length of each mouse at current time 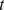 are initialised using previous frames’ estimates.
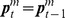


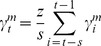
where 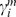 is the length of the mouse’s 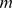 major axis at frame 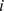 (which computation is described in the *M* step below), 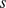 is the number of frames in the time window, and 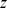 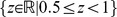 is a constant employed to make the system robust either to the displacement of mice between frames due to mouse movement or to the variable mouse length due to its non-rigid shape.

**Expecatation** ***(E)*** **:** the new mouse shape estimates are therefore re-computed using the standard watershed segmentation algorithm [41], starting from the current estimates of the background seed and the mice seeds determined at previous *M* step or during initialization. The result of this iteration is a binary image containing the shape of each single mouse 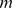.

**Maximization** ***(M)*** **:** a new estimate of the parameters is calculated from the mouse shapes in the binary image detected at previous *E* step. Specifically, 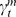 is the length of the major axis determined by the eigen analysis of the covariance matrix of the second order central moments of the mouse shape [40]. The mice position vector 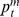 is therefore determined assuming the nose and the genitals at the two edges of the major axis. The orientation is determined on the basis of the Euclidean distance to the previous frame. The procedure converges when the estimated mouse shape is stable across the *EM* iterations.

The parameters of the very first frame (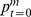 and 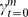) are estimated directly on the output of the Blob Detection module by analysing the major axis of each blob (with a similar technique described for the maximization step). As such, the first frame requires all mice to be disjointed for a correct estimation. If this is not the case, the algorithm skips the frame.

An automatic sanity check on the nose/genitals association is further performed to correct possible inversions. Similarly to the method proposed in [7], we exploit the direction of movement to correct the nose/genitals orientation. Our method, however, rather than associating the orientation at each frame, it needs to be corrected only in very few occasions, as the temporal watershed already provides the correct orientation with very high probability. For this reason, we performed this correction with a simpler and faster approach. Specifically, the sanity check module computes a directionality correction index 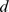, considering the directionality of the last 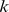 consecutive frames in a voting framework.
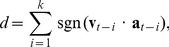
(2)where 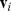 is the movement vector from frame 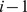 to frame 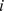 and 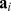 is the mouse direction, i.e., a vector that goes from the genitals to the nose. When the vote 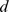 is negative, the head and tail positions are inverted in 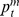. This sanity check guarantees stability in time, allowing the nose/genitals inversion only after a sufficient number of frames showing disagreement between mouse orientation and direction of movement. In our experiments we assumed 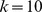.

### Mice Matching

Identity preservation is a major problem for any multiple animal tracking system [21], the difficulties lie on the resemblance between the animals, the absence of any type of visual tag and their continuous interactions causing occlusions. However, when mice are well separated, a properly contrasted video in conjunction with the temporal watershed approach allow good mice identity persistence based only on the Euclidean distance between 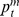 vectors in the current and previous frame. The order in which the distances are evaluated is crucial for a robust mice assignment. For each frame 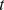, an adjacency matrix 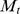 between mice positions at frames 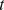 and 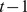 is created, with the mice distances as edges:
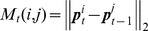
(3)


Hence, a greedy search [42] (using a minimum distance principle) is performed on this matrix to assign the mouse identifiers to their most probable shapes. This method is an approximation of the Hungarian algorithm, also used in [7], which substantially gives the same results when few entities need to be associated. Actually, we observed that the only mismatches appear when two mice are very near each other and aligned in the same direction becoming confused, like with the “Above” behaviour.

After the identity assignment, a further automated sanity check is performed to reveal possible mistracking due to mice proximity. This check is based on a metric defining the quality of results from the temporal watershed segmentation and it is the trigger to switch to the heat signature matching strategy. It was noticed that the vast majority of shape mistracking was due to a mouse seed trapped in a local minimum with the neighbouring seed overflowing on it. Therefore, the system monitors the evolution of the mice shapes and sets a misdetection flag whenever two or more mice significantly deviate from the “standard” shape distribution. Specifically, each mouse shape is described by the first Hu moment (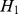), a single scalar value characterising a shape independently from translation, scale and rotation transformations [40]. The Student’s *t* distribution is then used to estimate the 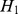 distribution for all shapes from frame 1 to frame 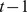. The misdetection flag is set thanks to a Student t-test whenever the null hypothesis of having the Hu moment not deviating from the baseline shape is rejected with a significance level below 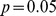.

In order to automatically deal with this misdetection, we developed a complementary method based on heat analysis that attempts to maintain mice identities when their geometrical characteristics are not suited for distance-based mice matching. The idea is to exploit the high sensitivity of the thermal camera used to record the video. Specifically, we use the temperature distributions over a small number 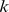 of frames in the video sequence, cumulated inside the mice shapes detected by the temporal watershed in the last 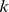 frames. This provides a dynamically changing temperature distribution for each mouse body. In our experiments, the size of a buffer 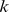 roughly corresponds to 0.3 seconds worth of video. At each new frame, the temperature distribution of each mouse in the current frame is compared to the heat signatures (temperature distributions determined in the moving time window) with the two-sample Kolmogorov-Smirnov test [43]. This test has multiple advantages. It does not make any assumption about the underlying distribution, it is fast to compute and it works directly on the samples. All the test results are stored in an adjacency matrix, and a greedy search similar to the one previously described is employed for mice assignment correction.

### Automatic Social Behaviour Classification

Once mouse positions, shapes and identities are tracked, the task of classifying their social interaction is undertaken. A classifier is trained to recognize the different behaviours on a set of examples provided by the expert as labelled videos. The action of each mouse in each frame of new videos (never seen by the system) is then automatically detected as belonging to one of the behavioural classes learned by the classifier. This is made possible by a proper representation of all possible mouse behaviours through spatio-temporal features, followed by the analysis performed with a random forest classification approach [25], which has been extended to exploit temporality as well.

To make the system scalable to a variable number of mice, all social behaviours are decomposed into all possible pairwise interactions, with a reference mouse that actively performs the action and a target mouse that is subjected to it. This considerably simplifies the definition of the feature vector and the further analysis of mice behaviour, since it has to deal only with the pairwise interactions of all mice pairs. All pairwise interactions are therefore opportunely combined after the separated classifications in order to have a single behaviour for each mouse at any given time-point (video frame).

### Spatio-Temporal Features

In order to capture the various aspects of all possible mouse interactions, the feature vector needs to describe relative position, movement and attitude of mice for all possible pairs. We propose a feature vector 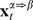 for each mouse pair 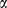 (the reference mouse) and 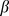 (the target mouse) as a set of spatio-temporal features specifically designed to capture the interactions at time frame 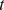 from video recorded with overhead cameras. As shown in Fig. 2, the feature vector is composed of 13 measurements, which can be divided into three main categories:

**Relative Position:** the Euclidean distances between the two mice measured between the corresponding three key points identified by the position tracker try to capture the relative static behaviours. Specifically, four distances (“head2head”, “head2body”, “head2genitals” and “genitals2genitals”) are able to unambiguously describe relative positions, such as side by side or nose interactions. A further “body2body” distance is computed as the distance of the reference mouse from the closest one. Differently from all other pairwise features, which are computed between the reference and target mice, this one is computed between the reference and the nearest mice, with the aim to better highlight social and non-social behaviours of the reference mouse.

**Shape:** two features containing the first Hu moment (previously described) and the area of the reference mouse attempt to capture the “attitude” of the mouse, such as being active, sleeping, exploring, etc.

**Movement:** the two previous feature categories capture a snapshot of the mouse actions in a frame. However, they are not representative of the action dynamics. This is accomplished also considering the movements of the reference mouse in a temporal window covering the past and the future of current timeframe. The Euclidean distance between the mouse centroid at frame 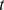 and the corresponding centroids at frames 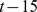, 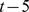, 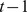, 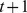, 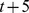, 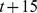 allows a multi-scale estimation of speed and acceleration of each mouse. The time offsets were empirically chosen on the basis of observations of mice movements in the training set.

### Temporal Random Forest Classification

In the proposed activity classification module we extended the random forest (RF) approach to the multi-frame case, performing a temporal evaluation of the tree ensemble, which provides the continuous and temporally regularised action description of the mice. This approach differs from the existing RF-like methods as it allows class predictions by generating multiple decision trees modelling the feature vector evolution through time. At the same time, it supports regularisation of the estimated behaviours avoiding abrupt changes of the predicted class. This framework is applied separately to each possible mice pair, represented with the feature vector 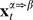. All feature vectors are independently classified, and the outputs are combined in order to obtain a continuous labelling of behaviour for each mouse in the video (see Fig. 2). Finally, the mouse behaviours can be analysed along all the video duration, either globally or in their time evolution. More specifically, the behaviour can be analysed as either summarizing the total time spent by each single mouse or by the group in a specific behavioural condition or, alternatively, counting the number of occurrences. In all our experiments we always used the total time.

In the proposed solution, 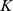 random trees 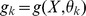 are built for each frame using labelled videos
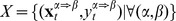
(4)where all feature vectors 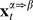 are projected in a random subspace 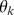 (i.e., represented by different random subsets of features) and are labelled with a behaviour class 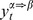. No pruning is performed. At each branch, the decision was based on the *information gain*. Every decision tree 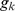 is inherently multi-class and it casts a vote for one of the behaviour classes learned during training. All random trees are then combined to form an ensemble (forest) used to associate a behaviour to each mouse in each frame of the testing videos with a voting strategy. Specifically, given a new feature vector 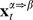 the votes of the random forest at frame 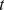 is defined as the set of votes of all random trees



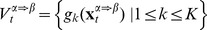
(5)The behaviour of the mouse pair 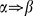 is obtained by further extending the forest with decision trees determined across neighbouring frames as follows:
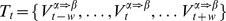
(6)where 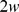 is the size of the time window surrounding frame 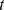, and 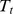 is the overall ensemble of votes for frame 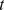. The activity classifier 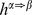 is therefore obtained as the *mode* of the set of votes, i.e. the most frequent behaviour class:



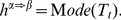
(7)The advantage of using this temporal Random Forests framework is twofold. On one side, the statistical robustness of the classifier is increased thanks to the ensemble of classifiers in the time dimension, which acts as a regularizing factor toward the optimal estimator for the current window. On the other side, the ensemble of decision trees in the temporally shifting window is trained considering the label of the central frame. However, each single tree in the forest exploits the different perspective on the data (i.e., the different underlying features). In this way, the ensemble exploits both past, present and future information to predict the label of the central frame.

In all our experiments we made forests of 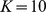 trees for each frame in a window of radius 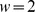, and projecting the data into subspaces of dimension 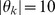.

Note that 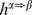 could be trained on a representative video segment on one mouse pair only while being exploited to classify the behaviour of all possible 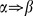 combinations in any video.

Given 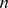 mice in the arena, there are 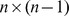 possible mouse pairs, 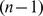 of which are those involving a specific mouse as the “reference mouse”, that is 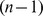 possible behaviour classes for each mouse. Nevertheless, for intelligibility, the system is expected to produce only one class label for each mouse at every frame 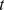. This issue is solved designing a priority table (see Fig. 1, where small numbers mean higher priority) indicating the “preferred” behaviour class among the available multiple choices, i.e., the one with the highest priority is assumed as the final class label. For example, assume a situation with three mice: for mouse 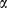 there are two behaviours determined by the system 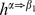 and 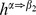. If 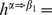 “Nose2Body” and 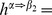 “StandAlone”, the behaviour of mouse 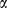 will be classified as “Nose2Body” because of its higher priority.

We compared our classification approach with one of the most recent method for mice behaviour analysis proposed in literature that also use a machine learning perspective, i.e. the one employed by Burgos-Artizzu et al. [23]. They employ a multi-stage classification approach based on an extension of the auto-context [13] (temporal context features) and multi-class AdaBoost. While in the original paper a mixture of Cuboids+Pca-Sift and trajectory features were chosen, we employed their classification strategy with our set of Spatio-Temporal Features. We obviously added the derived temporal context features to the data representation for the auto-context AdaBoost method. This allowed us to fairly compare the two different computational frameworks, which are both able to manage the temporal information in the data. A further extension of our feature set with the full-frame features used by Burgos-Artizzu et al. was not possible since they are meaningful only when two mice are in the arena and only one of the two is considered active. On the contrary, when more than 2 mice are considered, we need to classify the behaviour of each mouse separately using a different representation for each different mouse for each time instant.

## Results

### Evaluation of Position Tracking

We first evaluated the position tracking precision, particularly in relation to mouse identities interchanges. Table 1 shows the average number of manual interventions required to maintain the mouse identities in a sample of 30 minutes of video tracking in a cage of 3 mice (“dataA-1”). The complete tracking system, combining the distance-based matching and the heat-based matching, requires an average of 0.8852 manual intervention every 30 seconds. This is a considerable improvement in comparison to the two matching strategies by themselves, which achieved 1.2623 (distance-based matching) and 6.0328 (heat-based matching) manual intervention respectively. Note that the distance-based matching and the heat-based matching concurrently fail only 24% of times, allowing further improvements of the tracking performance by tuning the matching selection strategy.

**Table 1:** Number of manual interventions every 30sec used to correct the identities exchange od the tracking algorithm on dataset A.

	avg	std
Described System	0.8852	1.082
Distance Matching Only	1.2623	1.413
Heat Matching Only	6.0328	3.16

### Evaluation of Social Behaviour Classification

The choice of the training data is essential for a fair evaluation of the system. In all our experiments we adopted a strict 3-fold cross validation approach, i.e. the video is divided into three consecutive folds (leaving the frame ordering intact). A fold is used for testing and the other two are joined together to form the training set. This process is iterated over all folds. The same approach is used even when the classifier is trained on a mouse pair and tested on another, or when the classifier is trained on a grader and tested on the other grader. This avoids any chance of testing bias due to “double dipping” [44].

Dataset A was used to perform a quantitative analysis of the classification discrepancies among human graders and a comparison with the automatic system at a frame-by-frame level. This level of granularity allows being conservative in the evaluation. Table 2 shows the behaviour classification agreement among two human graders (top section) and between graders and automatic system (middle and bottom sections, respectively). In this table, all pairwise interactions are considered allowing a performance analysis before the combination of all pairwise classifications via the priority table to obtain unique behaviour for each mouse. Each value represents the percentage of frames showing a behaviour agreement in terms of accuracy and precision. High values indicate that a great number of frames have been annotated with the same behaviour. The results are further divided into sub-columns to better evaluate the agreement for different groups of behaviour: social and non-social (as shown in Fig. 1). In the classifier/grader comparisons, the classifier is tested for each grader with a 3-fold cross validation scheme. The average performance of all pairwise classifiers (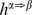) is also given, showing a grader/system’s consistency in line with the inter-grader one, i.e. the performance of our automated system is comparable to that of a human grader.

**Table 2:** Behaviour agreement among the two graders (top section) and quality of the system compared to the two graders (middle and bottom sections) on Dataset A (higher values are better ).

	*Among Graders*			
	*Acc. Full* ★	*Acc. SocVsNsoc* †	*Prec. Soc* ‡	*Prec. Nsoc*°
**dataA-1_redVsBlue**	80.68%	99.72%	97.36%	99.80%
**dataA-1_redVs** **Yellow**	80.29%	99.41%	88.39%	99.61%
**dataA-1_yellowVsRed**	89.31%	99.08%	97.65%	99.13%
**dataA-2_2Vs1**	69.91%	99.18%	98.92%	99.19%
**dataA-2_2Vs3**	70.18%	99.96%	99.29%	99.99%
**dataA-3_2Vs1**	72.94%	99.92%	98.36%	99.96%
**dataA-3_2Vs3**	72.90%	99.98%	99.71%	99.99%
**dataA-4_2Vs1**	66.57%	99.50%	91.26%	99.79%
*average*	*75.35%*	*99.60%*	*96.37%*	*99.68%*
	***System Vs. Grader 1***			
	*Acc. Full* ★	*Acc. SocVsNsoc* †	*Prec. Soc* ‡	*Prec. Nsoc*°
**dataA-1_redVsBlue**	76.16%	97.60%	72.00%	98.24%
**dataA-1_redVs** **Yellow**	78.01%	98.69%	74.85%	99.00%
**dataA-1_yellowVsRed**	81.61%	97.15%	68.22%	98.20%
**dataA-2_2Vs1**	69.94%	98.33%	92.36%	98.49%
**dataA-2_2Vs3**	70.94%	99.11%	95.91%	99.24%
**dataA-3_2Vs1**	73.09%	99.55%	95.16%	99.64%
**dataA-3_2Vs3**	73.71%	99.53%	96.55%	99.62%
**dataA-4_2Vs1**	72.06%	99.57%	97.56%	99.63%
*average*	*74.44%*	*98.69%*	*86.58%*	*99.01%*
	***System Vs. Grader 2***			
	*Acc. Full* ★	*Acc. SocVsNsoc* †	*Prec. Soc* ‡	*Prec. Nsoc°*
**dataA-1_redVsBlue**	85.46%	97.76%	76.56%	98.22%
**dataA-1_redVs** **Yellow**	86.33%	98.66%	84.44%	98.76%
**dataA-1_yellowVsRed**	85.37%	97.05%	58.40%	97.99%
**dataA-2_2Vs1**	77.01%	99.41%	89.96%	99.73%
**dataA-2_2Vs3**	75.73%	99.62%	93.57%	99.91%
**dataA-3_2Vs1**	79.92%	99.00%	73.40%	99.74%
**dataA-3_2Vs3**	79.60%	99.02%	80.67%	99.68%
**dataA-4_2Vs1**	91.98%	99.03%	86.79%	99.45%
*average*	*82.67%*	*98.69%*	*80.47%*	*99.18%*

The agreement is computed on a frame-by-frame basis. Each value represents the percentage of frames with class agreement. The different columns show the results for different types of behaviours:

★ accuracy on all behaviours considered separately;

† accuracy on behaviours grouped into social and non-social meta-classes;

‡ precision on the social behaviours;

°precision on the non-social behaviours. Accuracy = (TP+TN)/(TP+TN+FN+FP) and Precision = TP/(TP+FP) where TP = True Positive, FP = False Positive, FN = False Negative and TN = True Negative. The average agreement grader/system is comparable to the average grader/grader agreement.

Table 3 shows the above evaluation from a different perspective. Instead of measuring the frame-by-frame agreement, this table shows the behaviour duration agreement. This is achieved by calculating the average duration error for social and non-social behaviours in the grader/grader and grader/classifier cases. In all instances the time difference accounts for a few seconds only, thus highlighting the good performance of the algorithms. This kind of analysis elicits the dimension of the error that might affect the standard measures exposed by the experts to describe the behavioural phenotypes characterizing the mice strands under analysis.

**Table 3:** Average time difference (in seconds) between the overall duration of each behaviour as classified by the graders or by the system (less is better).

	*Among Graders*	*System Vs. Grader 1*	*System Vs. Grader 2*
**dataA-1_redVsBlue**	1.3 sec.	13.7 sec.	16.4 sec.
**dataA-1_redVsYellow**	2.3 sec.	8.7 sec.	15.0 sec.
**dataA-1_yellowVsRed**	10.7 sec.	8.8 sec.	13.5 sec.
**dataA-2_2Vs1**	12.0 sec.	20.2 sec.	1.2 sec.
**dataA-2_2Vs3**	0.4 sec.	9.3 sec.	3.3 sec.
**dataA-3_2Vs1**	0.0 sec.	4.7 sec.	9.1 sec.
**dataA-3_2Vs3**	0.0 sec.	5.1 sec.	6.9 sec.
**dataA-4_2Vs1**	2.3 sec.	7.1 sec.	2.6 sec.
*average (in sec.)*	*3.6 sec.*	*9.7 sec.*	*8.5 sec.*
*average (in percentage)*	*0.1%*	*0.3%*	*0.3%*

Social and non-social behaviours are grouped together. These values show a comparable time consistency between the system/grader and the two different human graders.

**Figure 4:**
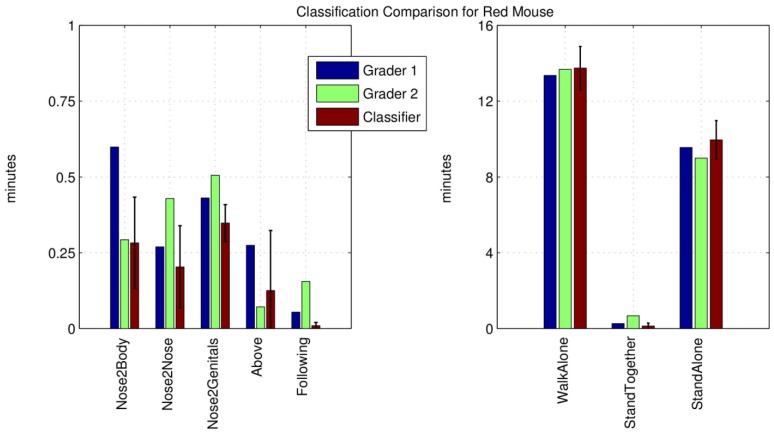
Overall mouse behaviour for one mouse in Dataset A-1 expressed in total time, as specified by the two human readers and automatically generated by our system. The results obtained by the classifier are comparable to those of the human scorers.

Fig. 4 shows the duration of each behaviour in the 30 minutes video for a sample mouse in dataset “dataA-1”, marked by the system with a red colour (see Fig. 3). In this case, the interactions are not limited to an average over all pairs, but the different classification are combined as described above, generalizing the result for the reference mouse against the other two mice. The first two columns show the behaviour durations estimated by the two human graders, the third column describes the average of the classifiers output trained on all other different mice pairs for both graders. The correlation between manually and automatically classified behaviour is substantial for the majority of the behaviours, with an exception for the behaviour “Following”, which is affected by a poor representativeness of in the training dataset, i.e., there are too few examples in the dataset for a good learning and a robust statistical estimation.

As previously discussed, we also compared our classification approach with the one proposed by Burgos-Artizzu et al. [23]. This work employed a multi-stage classification approach based on an extension of the auto-context [13] (temporal context features) and multi-class AdaBoost. Table 4 shows a summary of the comparison among the two classification strategies (the detailed ones are available as Additional Material): the tests are performed for all the behaviours learned from Dataset A and the results show that, in all instances, our approach outperforms [23] with any adopted measure of performance (accuracy, precision and time evaluation measurements).

**Table 4:** Comparison of classification performance between the method presented in this paper and the classification approach of Burgos-Artizzu et al. [23].

		*Temporal Random Forests (this paper)*	*Burgos-Artizzu et al.* [*23*]
***Grader 1 sets***	*Acc. Full*	*74.44%*	72.26%
	*Acc. SocVsNsoc*	*98.69%*	94.29%
	*Prec. Soc*	*86.58%*	75.11%
	*Prec. Nsoc*	*99.01%*	98.60%
	*Avg time diff.*	*9.7 sec.*	64.7 sec.
***Grader 2 sets***	*Acc. Full*	*82.67%*	78.60%
	*Acc. SocVsNsoc*	*98.69%*	94.28%
	*Prec. Soc*	*80.47%*	63.01%
	*Prec. Nsoc*	*99.18%*	98.83%
	*Avg time diff.*	*8.5 sec.*	61.2 sec.

The results are the average across all the mouse pairs of Dataset A, computed employing all the metrics of Table 2 and 3. In all instances our approach outperforms [23]. The full results are available as additional material (Table S1).

### Validation in Large Scale Studies

In the previous experiments comparing the system performance with a ground truth, the combination of classifiers trained on different mice pairs and graders showed excellent results as shown in Tables 2 and 3. Therefore, we used the trained system to analyse a larger dataset (Dataset B), which comprises of 9 hours of videos of freely interacting mice in groups of 2 or 4 in an open field arena. In the following results we decided to compare two mice strands observing the overall total amount of time spent in each behavioural condition, as it was sufficient to verify the starting hypothesis of socially impaired mice.

**Figure 5:**
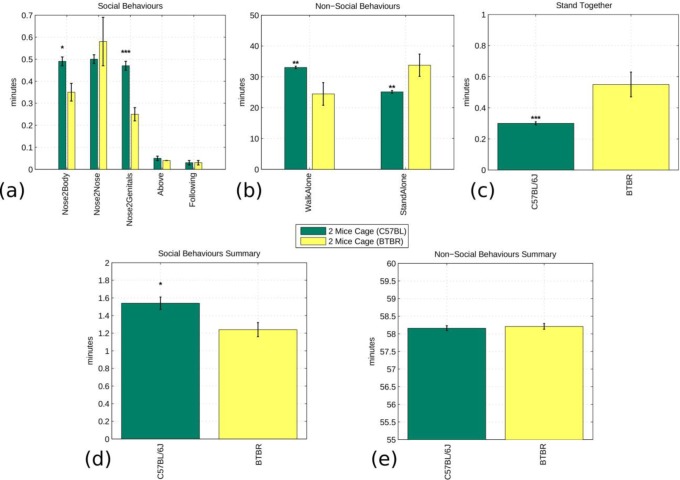
Fully automated analysis comparing the interactions of C57BL/6J (N = 10) and BTBR (N = 6) mice of all experiments with two animals per cage (Dataset B). The top graphs show the overall occurrence of each social and non-social interaction. We generated a different graph for “StandTogether” behaviour since its classification as either social or non-social is arguable. The bottom graphs show the aggregate comparison of social and non-social interaction. The null hypothesis that the C57BL/6J mouse and BTBR mice show a similar social/non-social behaviour is rejected in both cases by a two-class, two tail t-test that assumes equal variance. This shows impaired social activity in the BTBR case. The significance values of the t-test are (*) p<0.05, (**) p<0.005, (***) p<0.0005.

We first compared, the social and non-social behaviours of groups of BTBR T+tf/J (BTBR) and C57BL/6J (B6) mice. BTBR is a strain with established low sociability, while C57BL/6J (B6) has a standard social behaviour [37], [45]. Fig. 5 shows the comparison of the single behaviours for the two strains. BTBR male cagemates showed a significant decrease of “Nose2Body” (P<0.05) and “Nose2Genital” (P<0.0001) sniffing interactions compared to B6 mice. In accordance to this, BTBR male cagemates also showed a significant increase in the “StandAlone” behaviour (P<0.005; Fig. 5(b)) and appeared to be less likely to walk alone (P<0.005; Fig. 5(b)) compared to B6 mice. Overall, the sum of the single social and non-social behaviours demonstrated that our system was able to detect a significant reduction of social behaviours (P<0.05; Fig. 5(c)) and increase in non-social behaviours (P<0.05; Fig. 5(d)) in the BTBR compared to the B6 strain. This result is in line with what is already observed in other studies [37], [45].

**Figure 6:**
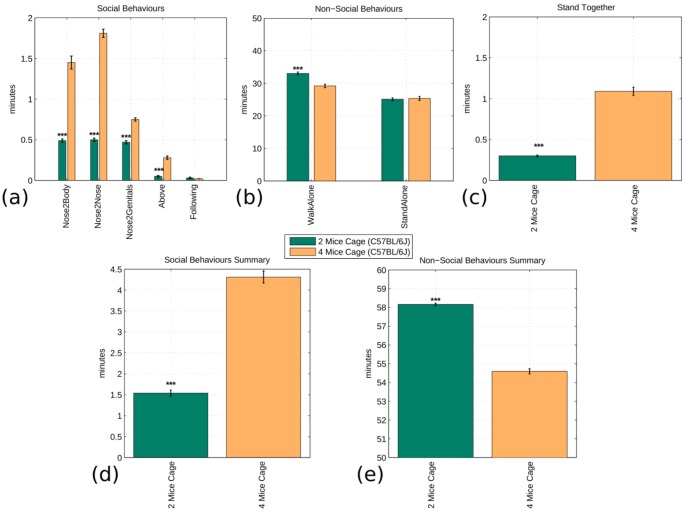
Fully automated analysis comparing the interactions of C57BL/6J mice in cages of two (N = 6) or four (N = 8) cage mates (Dataset B). The top graphs show the occurrence of each social and non-social interaction. For the same reasons exposed in Fig. 5 the “StandTogether” behaviour is shown in a separate graph. The bottom graphs show the aggregate comparison of social and non-social interaction. The null hypothesis that mice show a similar social/non-social behaviour, regardless of the number of mice interacting, is rejected in both cases by a two-class two tail t-test that assumes equal variance. This shows an increase of social activity when C57BL/6J mice can interact with more littermates. The significance values of the t-test are (*) p<0.05, (**) p<0.005, (***) p<0.0005.

In the last set of experiments, we compared the social interaction of an identical breed (i.e. B6) with 4 or 2 mice in the same arena. The system was able to reliably deal even with 4 mice simultaneously interacting in the same arena. Moreover, from Fig. 6 it was evident that, as expected, almost all social behaviours were increased when in the same arena there were 4 instead of 2 mice (P<0.0001; Fig. 6(a)). Similarly, there was a significant decrease of the “WalkAlone” behaviour in 4-mice cages (P<0.0001). In Fig. 6(c) and 6(d), the expected behavioural pattern is shown, i.e. social interactions in the 4 mice arena are increased (P<0.0001) while non-social behaviours are decreased (P<0.0001).

### Computational Performance

In our experiments, the position tracking algorithm runs in real time on a single core at 2.6GHz with a C++ implementation. A single frame is processed in ∼30 ms. The behaviour classification is performed offline, however the computational resources to generate the feature vectors and perform the classification are negligible.

## Discussion

In this paper, we addressed the challenging problem of multiple mice behaviour analysis devising a tracking/classification system, which presents several novel contributions. First, a real-time model-based segmentation and tracking algorithm that combining position, temperature and shape information is able to manage multiple interacting mice regardless of their fur colour or light settings is presented. A new set of specialised spatio-temporal features, targeted to be general enough to represent various aspects of static and dynamic social behaviours, are introduced. Finally, such features are used as the input for a learning-by-example classifier, based on random forest, which was extended to deal with video sequences.

Using a set of videos annotated by experts as training data, we were able to classify mice behaviours with an arbitrary number of mice (compatibly with the cage size and the experimental protocols). Currently, even the most recent methods have not attempted to monitor the behaviour of more than two mice concurrently. This is a substantial limitation in studies involving complex social behaviours as grouped mice might interact differently when groups have more than two components, as normally happens during human social relations.

Our experiments showed promising results in several aspects. Using three interacting mice, we obtained a tracking performance in line with the one presented by De Chaumont et al. [22], 0.825 vs. 0.885 manual interventions every 30 seconds in favour of [22], but testing their system uniquely with two mice. Concerning behaviour classification, we showed a system/human classification discrepancy comparable or better than the one among two human graders, looking at both a frame-by-frame analysis (Table 2) and a global time analysis (Table 3).

Various metrics were employed for the evaluation of behaviour classification. In the case of all behaviours considered separately, the system/grader frame-by-frame analysis showed a concordance often higher than the one among graders. First column in Table 2 shows an average concordance accuracy of 74.44%–82.67% for the automatic system and 75.35% for the human graders. To put these results into perspective, Burgos-Artizzu et al. [23] report a concordance among graders of 70% and a system/grader concordance of 61.2% by combining two views. However, they employed a different experimental setup with different behaviour classes, hence these results are not directly comparable. For a fair comparison we evaluated the performance of their classification strategy (Adaboost with auto-context features) with ours (Temporal Random Forests). Table 4 shows the results of these experiments, where our approach consistently shows better performance (Table S1, which shows the full list of experiments, is available as additional material). In the last three columns of Table 2 and 3, we evaluate the concordance of social vs. non-social behaviours, i.e. all behaviours are grouped in two “super-classes”. The frame-by-frame analysis of Table 2 shows an average concordance accuracy of 98.69% for the automatic system and 99.6% for the human graders. The global time analysis of Table 3 shows a time discrepancy of 8.5–9.7 seconds for the automatic system and 3.6 seconds for the human graders. These results account for less than 0.4% of the total video time, thus showing an extremely interesting ability of the system (and the graders) in evaluating the total time of social and non-social behaviours.

We also validated our system on a large dataset (Dataset B, 9 hours worth of video) showing two important aspects: the generalisation ability of the classifier on new datasets (the algorithm training was carried out on dataset A) and the applicability of the system on real behavioural studies. These experiments also highlight the need for more training data of some behaviour classes, namely “Above”, “Following” and “StandTogether”, which were rarely represented in the “training” data. Future development of the system will consider this limitation.

This paper is one of the forerunners of the automatic social behaviour classification in mice. For this reason, we made our dataset and grader labelling publicly available in order to allow other research groups to evaluate and compare their tracking and classification algorithms on multiple mice in infrared light with ours. We feel that the ability to design social experiments going beyond the resident-intruder approach presented by Burgos-Artizzu et al. [23] and de Chaumont et al. [22] is a necessity for the advancement of the study of complex social behaviours. Indeed, while in a resident/intruder set-up, the social behaviours are chiefly aggressive and initiated by a single mouse, in a “cagemates” set-up, the social interactions are likely to be finer and ultimately more complex.

Additionally, the ability to constantly monitor the behaviours of multiple mice in real time (even in near darkness) will greatly increase the amount of information that can be acquired in each experiment. Importantly, our system was able to discriminate different mice interacting together without any use of tagging which may alter the natural behaviour of mice. Moreover, as this classification method is based on a learning-by-example paradigm, it can be extended to analyse many other types of social behaviours, as long as there are enough examples that can be properly expressed by the feature vector.

## Conclusions

With the increased interest of molecular biologists in characterising and monitoring mice social behaviour, there is a raise in the demand of automatic video analysis systems that are easy to use, accurate and detailed. In this paper, we presented a complete method for the automatic tracking and classification of mouse behaviours from thermal video sequences. Quantitative tests performed using several sets of data show that our automatic behaviour analysis system can be successfully trained by examples and that its performance are in line with the performance of a human grader. Importantly, this system is able to go beyond the tracking and behaviour classification of only two animals at once, opening new perspectives in social behaviour studies. Moreover, this system is not only limited to social behaviour analysis but its tracking module can also measure accelerations, positions, etc. as in currently available single mouse tracking software packages.

## Supporting Information

Table S1 **Full comparative evaluation of the classification strategies in terms of accuracy, precision and global time difference.**
(CSV)
